# Resuscitation after cardiac surgery awareness: an Egyptian national survey

**DOI:** 10.1186/s43057-022-00067-6

**Published:** 2022-02-07

**Authors:** Moslem Abdelghafar, Taher Abdelmoneim, Alaa Mohamed, Mohamed Abdalla

**Affiliations:** 1 Department of Cardiothoracic Surgery, Wythenshawe Hospital, Manchester, UK; 2 Department of Cardiac Surgery, National Heart Institute, Giza, Egypt; 3 Department of Cardiothoracic Surgery, El-Hussine Hospital, Al-Azhar University, Cairo, Egypt; 4 Department of Cardiac Surgery, Shebein El Kom Teaching Hospital, Shebein El Kom, Egypt

**Keywords:** Cardiac surgery, Resuscitation, Training, Education

## Abstract

**Background:**

Cardiac surgery patients have different resuscitative needs than other patients who experience in-hospital cardiac arrest; this was addressed in the guidelines. However, it is unknown how widely the guidelines are practiced, or a training protocol is followed in different cardiac surgery units in Egypt.

**Methods:**

A 21-question survey was created and included: Participant demographics, prevalence of cardiac arrest, cardiac arrest protocol, emergency resternotomy technique, training protocols. Survey was disseminated through social media messaging platforms during the period between November 2020 and January 2021.

**Results:**

Ninety-five responses were from 11 centres across Egypt. In total, 68.5% of the respondents were surgeons, 76.8% of participants were junior surgeons. For patients who go into VF after cardiac surgery, respondents would attempt a median of 3 shocks with only 24.2% commencing defibrillation shocks before external cardiac massage, whilst the majority initiating CPR immediately and performing emergency resternotomy in a median time of 10 min. In total, 56.8% would give 1 mg of adrenaline as soon as the cardiac arrest was established. If a surgeon was not available, only 36.8% of respondents would allow any trained personnel to perform the emergency resternotomy. Only 9.5% practice regularly on emergency sternotomies. Seventy-five percent think tailored training is important and staff should be oriented about it in the future.

**Conclusion:**

An action plan is required to improve the training of the junior surgeons regarding the Cardiac Advanced Life Support Protocol to implement it in a timely organised manner. This should be endorsed and audited by a national society or body by keeping a national registry and mandatory recertification.

## Background

Every year, over 250,000 patients have cardiac surgery in some 450 centres in Europe [1] and more than 400,000 patients undergo cardiac surgery in the USA at approximately 1200 medical centres [2–4]. During the past decade, there has been an increasing recognition that cardiac surgery patients have different resuscitative needs than other medical and surgical patients who experience in-hospital cardiac arrest. The special resuscitative needs of cardiac surgery patients were addressed in the 2021 European Resuscitation Council (ERC) Guidelines for Resuscitation in the section reviewing cardiac arrest in special circumstances and the 2020 American Heart Association (AHA) guidelines for cardiopulmonary resuscitation and emergency cardiovascular care in special situations [5, 6]. However, it is unknown how widely the guidelines are practiced, or a training protocol is followed in different units in Egypt. This national survey aims to identify the views and common practice of Egyptian cardiac surgery teams regarding resuscitation after cardiac surgery.

## Methods and materials

A 21-question survey was created (Table 1) based on the original survey used by the European Association of Cardiothoracic Surgery (EACTS) guidelines committee [7]. Questions included the following topics: Participant demographics, prevalence of cardiac arrest in the intensive care unit, cardiac arrest with ventricular fibrillation or non-shockable rhythm, emergency resternotomy technique, training and arrest protocols. The survey was modified to collect participants’ demographics; other questions remained the same as the original survey. Survey dissemination was targeted to staffing of cardiothoracic departments in various institutes through social media platforms such as mobile messaging applications and emails during the time period between November 2020 and January 2021; this was our preference due to COVID-19 pandemic to allow better reach and in line with social distancing national guidance. Ethics approval and informed consent have been waived by the institutional review board.

**Table 1:**
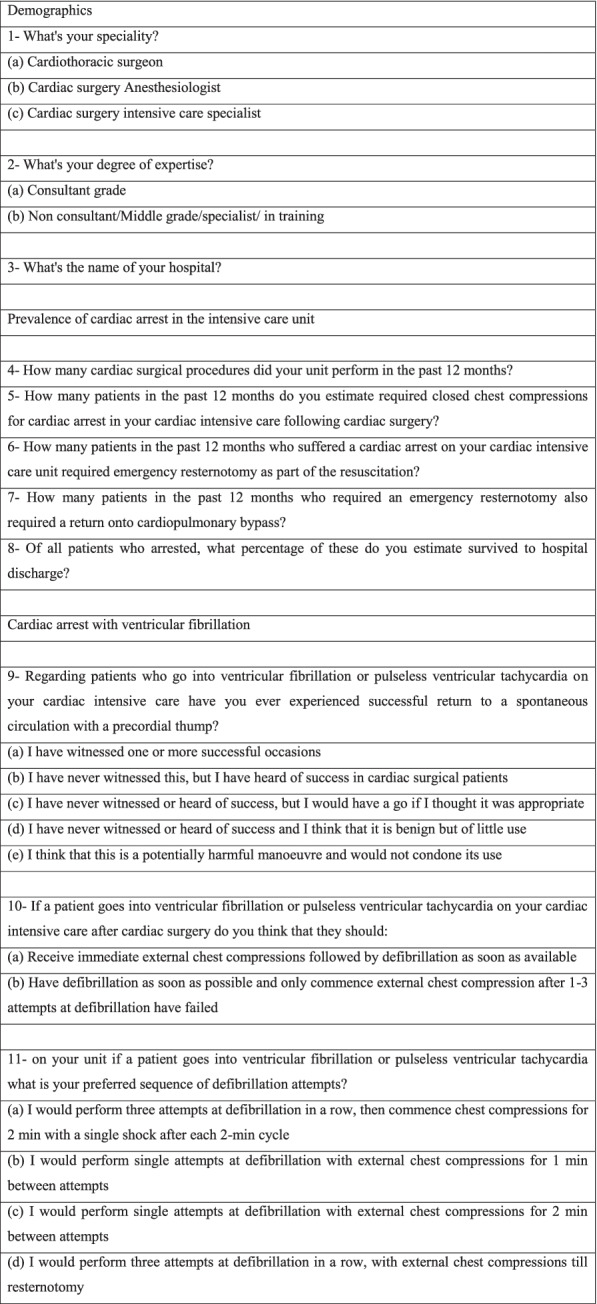

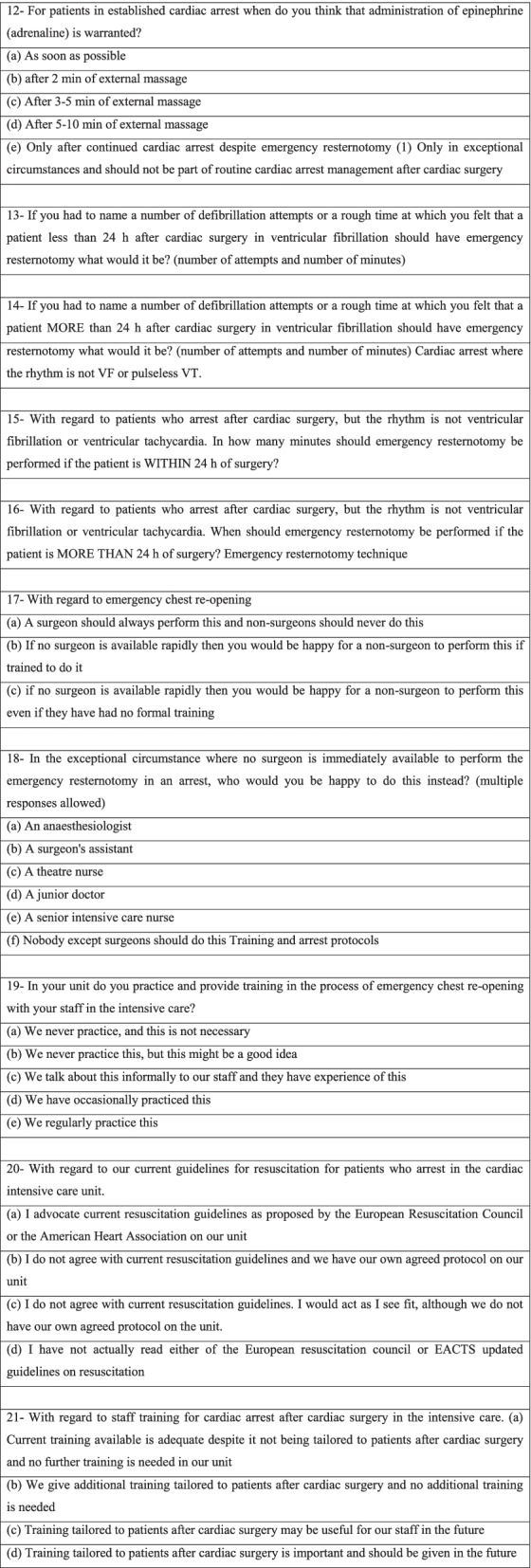
Depicting survey questions

### Demographics

The first eight questions were used to obtain demographic data on the expertise of the respondent, the size of the unit and the prevalence of cardiac arrest and emergency resternotomy in the unit where the respondent worked.

### Data cleaning

Prior to analysis, the data from all respondents were analysed independently and excluded if the multiple choice, numerical or text responses indicated that the survey had been incorrectly completed or if multiple respondents came from the same email address. Respondents were excluded if there were no responses to over 50% of the questions or if the numerical data responses were impossible (i.e. more arrests than operations performed in that unit).

### Statistical analysis

Continuous data are presented as median, mean, standard deviation and range or only as median if the data was significantly skewed using the Kolmogorov—Smirnov test. Categorical data was presented as percentages. Data was presented and analysed using SPSS 13.0 (Statistical Package for the Social Sciences, SPSS Inc., Chicago, USA).

## Results

Of 126 responses, 95 were suitable for inclusion. Thirty-one responses were deleted due to duplication or incorrect completion. We have responses from 11 centres across Egypt, 68.5% of the respondents were surgeons whilst cardiac anaesthetists and intensivists formed 12.6% and 18.9% respectively. The majority of participants were non-consultants/middle-grade doctors comprising 76.8%; consultant participation was 23.2%.

The median number of cases performed by all units was 480 and this ranged from 10 to 3000 annually. The average percentage of cardiac arrests in these units was 7%, and the average percentage of emergency resternotomy after cardiac arrest was 2.4%. Respondents reported that the median survival to hospital discharge of all arrests was 33%.

**Fig. 1:**
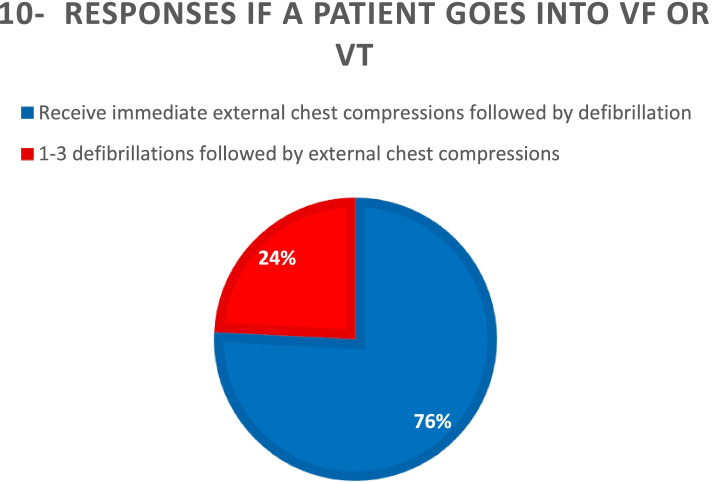
Percentage of respondents commencing ECM or DC shocks in a VF arrest situation

In patients who arrest with VF or VT, only 24.2% of respondents would commence 1‑3 defibrillation shocks and then perform external cardiac massage (ECM), with the majority initiating CPR immediately (Fig. 1).

**Fig. 2:**
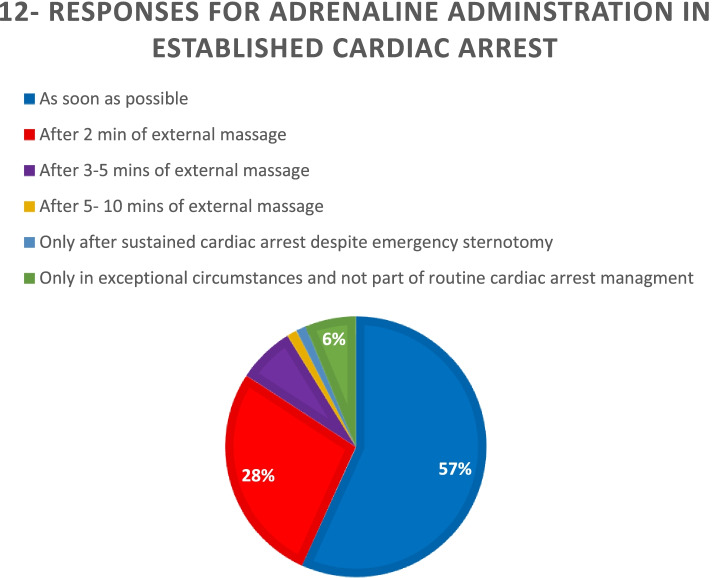
Adrenaline administration patterns after cardiac arrest

Regarding the sequence of defibrillation attempts interspersed with ECM, 50.5% of respondents would perform three attempts at defibrillation without intervening ECM. In total, 49.5% would perform single defibrillation attempts interspersed with ECM at 1- or 2-min intervals. In total, 56.8% of respondents would give 1 mg of adrenaline as soon as the cardiac arrest was established. Only 6.3% of respondents thought that it should be given rarely or not at all (Fig. 2).

For patients who go into VF in less than 24 h after cardiac surgery, respondents would attempt a mean of 3 defibrillation shocks and would perform emergency resternotomy in a median time of 10 min and in 15 min if the rhythm was not VF. Whilst for patients who suffer cardiac arrest more than 24 h after the surgery results were, a median of 5.6 attempts of defibrillation and a median of 17.6 min to perform emergency resternotomy (Table 2).

**Table 2:** Median of defibrillation attempts and time to perform emergency resternotomy

	Number of responses	Median	Range
Number of defibrillation attempts before resternotomy in VF/VT			
Cardiac arrest < 24 h	95	3 shocks	14 (min 1, max 15)
Cardiac arrest > 24 h	95	5 shocks	19 (min 1, max 20)
Time to resternotomy where initial rhythm is VF/VT			
Cardiac arrest < 24 h	95	10 min	29 (min 1, max 30)
Cardiac arrest > 24 h	95	17.6 min	58 (min 2, max 60)
Time to resternotomy where initial rhythm is asystole/PEA			
Cardiac arrest < 24 h	95	15 min	44 (min 1, max 45)
Cardiac arrest > 24 h	95	17.6 min	44 (min 1, max 45)

A series of questions were asked on the conduct of an emergency resternotomy in patients who have suffered a cardiac arrest (Table 3). If a surgeon was not immediately available, 36.8% of respondents would allow a suitably trained non-surgeon to perform the emergency resternotomy whilst 58.9% of respondents would not accept anyone except a surgeon to perform the resternotomy. Of respondents who would allow a non-surgeon to perform an emergency resternotomy, 33.7% would allow anyone trained for the procedure to do it, 14.4% would allow an anaesthesiologist, 17.9% would allow intensive care doctor and 12.6% would allow a junior grade doctor to perform an emergency resternotomy. Only 9.5% and 3.2% would allow a theatre scrub nurse and a senior intensive care nurse respectively to do this.

**Table 3:** A series of questions on the conduct of an emergency resternotomy in patients who suffered a cardiac arrest

	Responses	percentages
(6) Precordial thump (total)	95	
(a) Witnessed	36	37.9%
(b) Heard of success	15	15.8%
(c) Have a go	14	14.7%
(d) Of little use	18	18.9%
(e) Potentially harmful	12	12.6%
(7) Defibrillation or ECM for VF	95	
(a) Immediate ECM	72	75.8%
(b) Immediate defibrillation	23	24.2%
(8) Sequence of shocks for VF	95	
(a) Three attempts, 2 min ECM then single shocks	34	35.8%
(b) Single attempts with 1 min ECM	12	12.6%
(c) Single attempts with 2 min ECM	35	36.8%
(d) Three attempts, ECM till resternotomy	14	14.7%
(9) When is adrenaline warranted	95	
(a) As soon as possible	54	56.8%
(b) After 2 min of ECM	26	27.4%
(c) After 3‑5 min after ECM	8	8.4%
(d) Only after emergency resternotomy	1	1.1%
(e) Only in exceptional circumstances	6	6.3%
(15) Emergency resternotomy	95	
(a) A surgeon should always do this	56	58.9%
(b) A trained non-surgeon could do this	35	36.8%
(c) Any non-surgeons could do this	4	4.2%
(17) Do you train for emergency resternotomy	95	
(a) We never practise, not necessary	14	14.7%
(b) We never practise might be good idea	33	34.7%
(c) Informal talks and experience	11	11.6%
(d) We have occasionally practised	28	29.5%
(e) We regularly practise	9	9.5%
(18) Current guidelines for the ICU	95	
(a) I advocate the ERC/AHA 2005 guidelines	67	70.5%
(b) I do not agree with these, we have our own protocol	1	1.1%
(c) I do not agree with these, we have no protocol	4	4.2%
(d) I have not read the ERC/AHA guidelines	23	24.2%
(19) Current training	95	
(a) It is adequate currently but not tailored	17	17.9%
(b) We give additional training	7	7.4%
(c) Tailored training might be useful	18	18.9%
(d) Tailored training is important and should be given	53	55.8%

A total of 49.4% of the participants have never practised any local training to perform an emergency sternotomy; however, 34.7% believe it is a must-have. Forty-one percent of the respondents state they occasionally practice or talk with the staff about guidance in the event of a cardiac arrest. Only 9.5% practice regularly on emergency sternotomies.

Twenty-five percent assume that current training is sufficient and does not need modification or additional patient tailoring, whilst 75% think tailored training is important and staff should be encouraged to undertake it in the future.

In total, 70.5% of all respondents advocate the current guidelines for resuscitation published by the ERC, EACTS and the AHA for use on their patients; however, 5% disagree with the guidelines and have their own local protocol. Meanwhile, 24.2% have not read the guidelines.

## Discussion

The incidence of cardiac arrest after cardiac surgery is around 0.7‑7% [8–16], EACTS, ERC and AHA endorsed resuscitation guidelines for this special group. Our survey gives an insight into current practices and adoption of guidelines of the Egyptian cardiac surgery centres. To our knowledge, there were no studies on a national level to address post cardiac surgery resuscitation practices.

In our study, 68.5% of the respondents were cardiac surgeons, of which 76.8% middle grade/resident/junior doctors. This is representative of first responders to cardiac arrest call in a routine practice thus it is crucial to identify knowledge and practices to evaluate the quality and safety of patient care. However, we found no significant differences in practices amongst respondents from various institutions, consultant and non-consultants, surgeons and non-surgeons.

Our respondents will act in a VT/VF cardiac arrest situation as follows, 75% will start CPR, 57% will give adrenaline immediately. All of which are more in line with Advanced Life Support (ALS) or Advanced Cardiac Life Support (ACLS) protocols and not the protocol dedicated for cardiac surgery.

The current guidelines advocate, once cardiac arrest is identified, to assess the rhythm first and not to commence chest compressions, reason being the possibility of presence of shockable rhythm such as VF or pulseless VT in 25‑50% of cases. If a shockable rhythm is identified, chest compressions could be delayed for up to 1 min to deliver 3 shocks as this might spare the traumatic chest compressions to a fresh sternotomy wound and avoid complications of cardiac/graft injury [17, 18].

In the cardiac surgical patient, the efficacy of defibrillation reduces by 10% for every minute delay, in addition, success rates for immediate sequential shocks for VF or pVT decline from 78% with the first shock to 14% with the third, therefore, immediate defibrillation with three sequential attempts at 150 Joules is advised [19]. Whilst in severe bradycardia or asystole, it is advisable before starting chest compressions to turn the pacing to emergency setting or DDD mode, 90 beats, maximum amplitude.

No study concluded benefit or harm of administering adrenaline during resuscitation of the postoperative cardiac surgical patient; however, the risk of administering adrenaline in conventional doses is with profound hypertension, bleeding or tearing of vessel anastomoses on return of spontaneous circulation (ROSC), which can precipitate catastrophic harm or further cardiac arrest [20]. Therefore, the recommendation to administer adrenaline is to be delayed until reversible causes of arrest are excluded and directed by a senior clinician experienced in their use. Adrenaline remains a useful drug in peri-arrest situations in smaller doses.

Of concern, almost 60% would not prefer anyone but the surgeon to perform a resternotomy, we believe the main culprit is medicolegal claims and pursuits. Nonetheless, 17.9% would allow intensive care doctors trained for emergency sternotomy to perform it in case of cardiac arrest. This stems from the fact that junior surgeons in many Egyptian cardiac units are allocated to manage cardiac intensive care, thus, being familiar with surgical problems and have enough skills to perform an emergency sternotomy.

In total, 70.5% of respondents advocate the current guidelines for resuscitation yet only 10% train regularly. Practicing protocol-based arrest management has been shown to reduce by 50% the time to chest reopening, reduce complications resulting from the resternotomy after cardiac surgery and improve survival [21–25]. Thus, the need to raise the awareness and training of the junior surgical doctors and intensive care staff with current guidance and emergency sternotomy protocols is paramount.

A structured national registry and regular auditing are key features to achieve the compliance, training, and monitoring of trainees; in addition, regular mandatory recertification is crucial to maintain an up-to-date knowledge of the current pool of surgeons and fellows. This could be endorsed by the cardiothoracic society body or national health service in the country.

The core message for our trainees and fellows; external chest compressions are ineffective in tamponade, extreme hypovolemia due to bleeding. Brain damage will occur in 5 min; the only way to save those patients is to perform a rapid smooth emergency resternotomy.

### Limitations

Our study has several limitations. Survey respondents are almost always self-selected, not everyone who receives a survey is likely to answer it despite offered incentives, which explains the small number of respondents. However, this was the best available alternative for multi-centre data collection due to COVID-19 pandemic and the national guidance for social distancing.

Data regarding the number of procedures, closed chest compressions, resternotomy and going back on bypass rates may not represent the actual figures and numbers as 77% of the respondents are middle-grade doctors and might not be able to access these institutional numbers readily. Moreover, COVID-19 pandemic has significantly impacted the number of procedures performed and could be a valid reason for the heterogeneity of data from respondents from the same centre.

## Conclusions

This is the first study on a national level to address post-cardiac surgery resuscitation, it revealed a gap in the knowledge and current practices. An action plan is required to train the junior surgeons on the Cardiac Advanced Life Support Protocol, which should be endorsed and audited by a national society or body through a national registry and mandatory recertification.

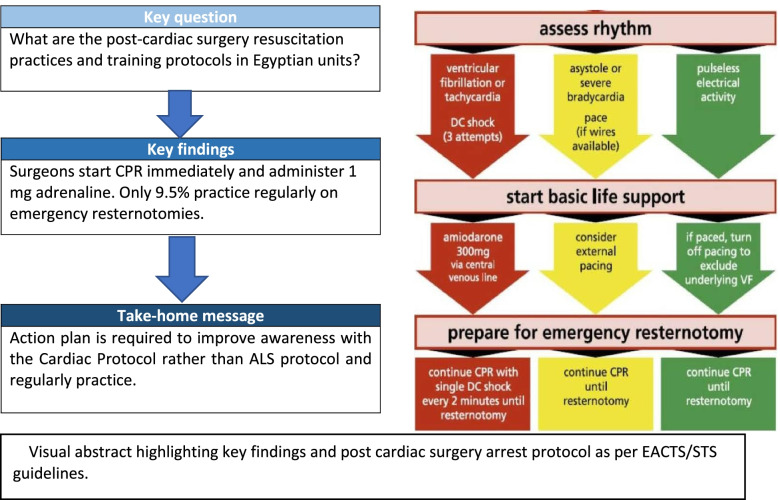



## Data Availability

The data sets used and/or analysed during the current study are available from the corresponding author upon reasonable request.

## Abbreviations

ACLS: Advanced Cardiac Life Support
AHA: American Heart Association
ALS: Advanced Life Support
CPR: Cardiopulmonary resuscitation
EACTS: European Association of Cardiothoracic Surgery
ECM: External cardiac massage
ERC: European resuscitation council
ICU: Intensive care unit
ROSC: Return of spontaneous circulation
PEA: Pulseless electrical activity
STS: Society of Thoracic Surgeons
VF: Ventricular fibrillation
VT: Pulseless ventricular tachycardia
